# Plastic Pirates Nova Scotia 2024 dataset - citizen science investigation of anthropogenic litter pollution of aquatic environments

**DOI:** 10.1016/j.dib.2025.112203

**Published:** 2025-10-20

**Authors:** Tim Kiessling, Maya Goldchtaub, Plastic Pirates of Nova Scotia, Sinja Dittmann, Janto Schönberg, Tony R. Walker

**Affiliations:** a Kiel Science Outreach Lab (Kiel University & Leibniz Institute for Science and Mathematics Education), Am Botanischen Garten 16i 24118 Kiel, Germany; b School for Resource and Environmental Studies, Dalhousie University, Halifax, NS B3H 4R2, Canada; c Direct Action Research Collective, Prof.-Anschütz-Str. 72 24118 Kiel, Germany; d Let’s Talk Science Dalhousie University Outreach Site, Dalhousie University, Halifax, NS B3H 4R2, Canada

**Keywords:** Plastic pollution, Plastic waste, Macrolitter, Schoolchildren, Participatory research

## Abstract

This dataset describes anthropogenic litter pollution, including plastic pollution, at nine sampling sites at lakes and the ocean in Nova Scotia in the fall of 2024. It comprises data from two different citizen science protocols. The first protocol (“Group A”) assessed litter density per m² and litter type (paper, cigarettes, plastic, metal, glass, food leftover, other). A total of 675 m² were investigated using this protocol, and a total of 495 litter items classified. The second protocol (“Group B”) assessed the litter item composition of individual items, based on 24 litter categories, including nine single-use plastics categories. In total 3032 litter items were collected and sorted into these categories, representing 114 kg of litter. In total, 20 sampling activities (Group A and B combined) were conducted at nine sampling sites. Potential sources of litter items were also evaluated, and each sampling site was described in detail, including coordinates, shape, slope and orientation of the shoreline and accessibility. The data were collected by 275 people from nine organizations, most of them citizen scientists. Collectively, they contributed 635 h of data collection effort, including litter collection, litter sorting and data annotation. Most of the citizen scientists were schoolchildren, aided in their research by their teachers and the coordinators of this project. The implementation of the project in Nova Scotia was part of the Plastic Pirates program (https://www.plastic-pirates.eu/), investigating litter pollution in different countries in cooperation with schoolchildren and teachers. This open access dataset, available on Zenodo (https://doi.org/10.5281/zenodo.16949607) [1] under Creative Commons license CC BY 4.0, can be used to compare litter densities and litter composition across regions in Canada or worldwide, and is of value as a reference data point in time to assess litter pollution in temporal litter studies.

Specifications Table

**Table:** 

Subject	Earth & Environmental Sciences
Specific subject area	Pollution of aquatic environments by anthropogenic litter, including plastic litter
Type of data	Raw data, Summarized data, Metadata, Tables
Data collection	Anthropogenic litter pollution was investigated in Nova Scotia in October and November of 2024 by employing two different citizen science protocols. The first protocol (Group A) required establishing transects and sampling stations at the shore with measuring tapes, tent pegs and ropes. The litter items within the sampling stations were collected and classified according to type. The second protocol (Group B) required collecting as much litter as possible along the shore within a certain time frame (depending on the number of citizen scientists and their available time). Subsequently, the litter was classified according to 24 different categories.
Data source location	Litter was collected between the 10th of October and the 16th of November 2024, at nine sampling sites (seven at lakes and two at the ocean) in Nova Scotia (Canada). In total, 20 sampling activities were conducted. The coordinates of sampling sites are included in the dataset.
Data accessibility	Repository name: Zenodo Data identification number: 10.5281/zenodo.16949607 Direct URL to data: https://doi.org/10.5281/zenodo.16949607
Related research article	none

## 1 Value of the Data

• These data provide information about anthropogenic macrolitter pollution at selected sampling sites in Nova Scotia. They therefore provide baseline information of litter density per m², type of litter, as well as litter composition of objects, including single-use plastics.
• These data are directly comparable to the international citizen science programs Plastic Pirates in Europe and Científicos de la Basura in Latin America and can be harmonized to be comparable to further international citizen science programs investigating anthropogenic litter pollution, such as the International Coastal Cleanup, Marine Debris Tracker, and Marine Litter Watch.
• The obtained data, when combined with other data sources, are valuable for researchers interested in temporal aspects of litter pollution or the evaluation of policy measures being implemented to combat anthropogenic litter pollution.
• The data further illustrate the value of research approaches involving citizen scientists and herein, mainly schoolchildren and their teachers, to investigate anthropogenic litter pollution. Data quality was ensured by using an established citizen science method and preparatory meetings before samplings. The employed protocols can be built on by scientists, citizen science coordinators and science communicators working at the interface of education and research.

## 2 Background

Anthropogenic litter pollution, and especially plastic pollution, is a pervasive and global problem, with profound environmental, socioeconomic and human health impacts [[2], [3], [4]]. Actors from the general public have been involved in investigating the extent of anthropogenic litter pollution in citizen science approaches over the past decades [5]. Examples include clean-up activities with accompanying data collection of collected litter items [6], and dedicated plastic pollution research programs involving schoolchildren [7]. These investigations do not only provide valuable research data but have the opportunity to foster the environmental awareness or scientific literacy of participants [8]. This article describes a dataset collected through the Plastic Pirates citizen science program involving schoolchildren and their teachers in Nova Scotia (Canada). Litter density and litter item composition were assessed at lakes and coastlines, employing two protocols focused on pollution by macrolitter (i.e. litter items larger than 5 mm), and especially plastics. The collected data can be used as comparison to other sampling sites (as the Plastic Pirates method is used in multiple countries).

## 3 Data Description

This article describes the dataset “Plastic Pirates in Nova Scotia – Litter pollution results for fall 2024”, available on Zenodo (https://doi.org/10.5281/zenodo.16949607) [1]. The dataset consists of three files:

• A spreadsheet file containing research data as well as metadata (“Plastic Pirates Nova Scotia 2024_Litter pollution dataset.xlsx”). The spreadsheet file consists of five datasheets and contains metadata related to the participating organizations and investigated sampling sites as well as the research data (Table 1, Table 4). The individual data fields of each datasheet are described in Tables 2–6.

**Table 1:** Description of the datasheets in the spreadsheet file “Plastic Pirates Nova Scotia 2024_Litter pollution dataset.xlsx”.

Name of datasheet	Description of datasheet
1_Metadata	Contains information about each individual sampling event such as sampling date and location, participating organizations, the number of participating schoolchildren and volunteers, sampling effort and time, and characteristics of sampling sites (lake or ocean sampling, urban development next to site, orientation of the shore, and accessibility). These descriptions refer to the entire sampling site, encompassing the locations where multiple sampling activities took place.
2_GroupA_Data	Contains research data about litter density and type of litter objects (Group A) for each individual sampling station positioned on transects (see the description of methods below).
3_GroupA_Overview	Provides an overview of the data of Group A, summarized for each sampling activity.
4_GroupB_Data	Contains research data about litter item composition (Group B) for each sampling site, according to 24 different categories, including single-use plastics (see the description of methods below). The approximate area in which litter was collected is also indicated.
5_LitterSources	Contains research data related to potential sources of anthropogenic litter encountered (see the description of methods below).

**Table 2:** Description of data fields of datasheet *1_Metadata*. The data is organized in rows, each row represents one sampling event.

Data field	Description
ID	A unique identifier for each sampling event.
Organization_Name	The name of the organization that participated in the project.
Organization_Type	The type of organization that participated in the project (school, university or non-governmental organization (NGO)).
SamplingLocation_CoordinatesGatheringPoint	The coordinates of the general sampling site, often the gathering point of the participants to conduct the sampling. The coordinates for the sampling conducted by St. Margaret's Bay Stewardship Association remain undisclosed due to nature conservation. The more precise coordinates for the sampling activity litter density and litter type (Group A) can be seen in datasheet *2_GroupA_Data* (also see Table 3).
SamplingLocation_NameOfCoast	The name of the shore of the lake or ocean site where the sampling took place.
SamplingLocation_NameOfWaterBody	The name of the lake or ocean basin where the sampling took place.
SamplingLocation_WaterBodyType	The type of the water body where the sampling took place (lake or ocean).
SamplingDate	The date of the sampling.
Participants_SchoolGrade	The grade of school the schoolchildren attended at the time of sampling (na = participants were not schoolchildren).
Participants_NumberOfSchoolchildren	The number of schoolchildren who participated in the sampling (na = participants were not schoolchildren).
Participants_NumberOfOtherCollaborators	The number of people besides schoolchildren who participated in the sampling, such as project coordinators, teachers and volunteers. Some people participated multiple times (mainly TK), they are counted once per sampling event!
Participants_NumberTotal	The total number of people who participated in the sampling, including schoolchildren and other collaborators.
SamplingTime	The approximate time the participants spent in the field, including the preparatory meeting, conducting the sampling activities and discussing results. Travel time was not included.
SamplingTime_CollectiveEffortInHours	The collective sampling effort contributed by the participants, i.e. the sum of total participants multiplied by sampling time; expressed in hours.
SamplingSite_CoastType	The type of coast the sampling took place at (urban, semi-urban or na = sampling activities that took place at the undisclosed location). Assessed through mapping and satellite imagery (OpenStreetMaps [9], Google Earth Pro).
SamplingSite_AdjacentEnvironment	A description of the environment adjacent to the sampling site (na = sampling activities that took place at the undisclosed location). Assessed through mapping and satellite imagery (OpenStreetMaps, Google Earth Pro).
SamplingSite_BuildingsOrDevelopmenAtAdjacentEnvironment	A description of buildings or infrastructure adjacent to the sampling site (na = sampling activities that took place at the undisclosed location). Assessed through mapping and satellite imagery (OpenStreetMaps, Google Earth Pro).
SamplingSite_CoastlineOrientation	The orientation of the sampling site, i.e. in which cardinal direction the sampled shore is facing (na = sampling activities that took place at the undisclosed location). Assessed through mapping and satellite imagery (OpenStreetMaps, Google Earth Pro).
SamplingSite_CoastlineCurvature	The curvature of the sampling site (concave, linear, convex, rugged, na = sampling activities that took place at the undisclosed location). Assessed through mapping and satellite imagery (OpenStreetMaps [8], Google Earth Pro).
SamplingSite_CoastlineSlope	The slope of the sampling site (none, gentle slope, moderate slope, steep slope). Assessed by the coordinator during the sampling.
SamplingSite_CoastlineAccess	The type of access to the sampling site (pedestrian only or vehicle and pedestrian access).
Note	Any note of interest regarding the respective sampling event.

**Table 3:** Description of data fields of datasheet *2_GroupA_Data*. The data is organized in rows, each rows represents a sampling station for the protocol litter density and litter type. See below for the description of the sampling protocol.

Data field	Description
ID	A unique identifier for each sampling event.
Sampling ID_LitterDensities	A unique identifier for this sampling activity (litter density and litter type).
Sampling_Coordinates	The coordinates of each transect at the sampling site (na = this station was not conducted due to reasons mentioned in the data field *Note*, it is listed for the sake of completeness).
Transect	The number of the transect on which sampling stations were positioned.
Station	The letter denominating each station on the respective transect (A, B or C).
Litter_Paper	The number of litter items consisting of paper found within the respective sampling station.
Litter_CigaretteButts	The number of cigarette butts found within the respective sampling station.
Litter_Plastic	The number of litter items consisting of plastic found within the respective sampling station.
Litter_Metal	The number of litter items consisting of metal found within the respective sampling station.
Litter_Glass	The number of litter items consisting of glass found within the respective sampling station.
Litter_FoodLeftover	The number of food leftover items found within the respective sampling station.
Litter_OtherWaste	The number of other waste items not belonging into any of the other categories found within the respective sampling station.
Litter_Total	The total number of litter items found in the respective station, i.e. the sum of the previous data fields.
AreaSampled_m2	The area sampled for each respective station in m², i.e. the size of the sampling station (= 9 m²). The number of investigated stations and transects, and therefore the area, depended on the available time of the citizen scientists.
Litter_Average	The average number of litter items found within the respective sampling station, i.e. the value of total litter divided by the area sampled.
Note	Any note of interest regarding the respective sampling station, e.g. the reason why a specific station could not be sampled.

**Table 4:** Description of data fields of datasheet *3_GroupA_Overview*. The data is organized in rows, each rows represents the summary of all stations conducted for the respective sampling activity. See below for the description of the sampling protocol.

Data field	Description
ID	A unique identifier for each sampling event.
Sampling ID_LitterDensities	A unique identifier for this sampling activity (litter density and litter type)
Paper_total	The total number of litter items consisting of paper found within all stations on all transects of the respective sampling site.
CigaretteButts_total	The total number of cigarette butts found within all stations on all transects of the respective sampling site.
Plastic_total	The total number of litter items consisting of plastic found within all stations on all transects of the respective sampling site.
Metal_total	The total number of litter items consisting of metal found within all stations on all transects of the respective sampling site.
Glass_total	The total number of litter items consisting of glass found within all stations on all transects of the respective sampling site.
FoodLeftover_total	The total number of food leftover litter items found within all stations on all transects of the respective sampling site.
OtherWaste_total	The total number of other waste items found within all stations on all transects of the respective sampling site.
GrandTotal	The grand total number of litter items found within all stations on all transects of the respective sampling site, i.e. the sum of the previous data fields.
AreaSampled_m2	The total area sampled for all stations on all transects of the respective sampling site.
Litter_Average	The average number of litter items found for all stations on all transects of the respective sampling site, i.e. the value of grand total litter items divided by the area sampled.
Note	Any note of interest regarding the sampling activity conducted at this site.

**Table 5:** Description of data fields of datasheet *4_GroupB_Data*. The file is organized in rows, each rows represents the data for each sampling activity. See below for the description of the sampling protocol.

Data field	Description
ID	A unique identifier for each sampling event.
Sampling ID_LitterComposition	A unique identifier for this sampling activity (litter composition)
SamplingArea_length_m2	The length of shoreline the citizen scientists covered to collect litter items. This value is a very rough approximation.
SamplingArea_width_m2	The width of shoreline the citizen scientists covered to collect litter items. This value is a very rough approximation.
SamplingArea_totalm2_approx	The total area of shoreline the citizen scientists covered to collect litter items, i.e. the sum of the sampling length and width. This value is a very rough approximation and should not be used to calculate litter density.
PlasticBags	The number of any plastic bags, paper bags containing plastics, and fragmented plastic bags (if they still could be identified as such) found at the respective sampling site. This category was considered to be a single-use plastic item category.
PlasticBottles	The number of any beverage plastic bottles found at the respective sampling site. Non-beverage plastic bottles were not included in this category. This category was considered to be a single-use plastic item category.
PlasticLids	The number of any beverage plastic lids found at the respective sampling site. Non-beverage plastic lids (for example lids of food containers) were not included in this category. This category was considered to be a single-use plastic item category.
PlasticTakeAwayFastFood	The number of any fast food container consisting entirely or partially of plastic (e.g. fast food beverage containers with plastic lining) and take-away container (including coffee to go cups and their lids) found at the respective sampling site. This category was considered to be a single-use plastic item category.
PlasticCutleryPlates	The number of plastic cutlery (including coffee stirrers and plastic straws), plastic plates, and plate-like plastic items found at the respective sampling site. This category was considered to be a single-use plastic item category.
PlasticSnackPackaging	The number of plastic packaging for snack foods (including packaging for sweets, crisps and savoury snacks) found at the respective sampling site. This category was considered to be a single-use plastic item category.
PlasticCottonBuds	The number of cotton buds with a plastic rod found at the respective sampling site. Cotton buds without plastic rod were not included in this category. This category was considered to be a single-use plastic item category.
PlasticHygiene	The number of hygiene products containing plastics (including tampons, wet wipes and tampon applicators) found at the respective sampling site. This category was considered to be a single-use plastic item category.
PlasticPolystyrene	The number of polystyrene items (excluding those categorized as PlasticTakeAwayFastFood), including polystyrene beads and broken packaging found at the respective sampling site.
PlasticCigaretteButts	The number of cigarette butts (excluding vapes and cigarette packaging) found at the respective sampling site. This category was considered to be a single-use plastic item category.
PlasticSmallPieces	The number of small plastic pieces regardless of shape, which could not be assigned to another category, found at the respective sampling site.
PlasticOther	The number of any other item consisting entirely or partially of plastic (e.g. tetrapaks) found at the respective sampling site.
MetalCans	The number of aluminum beverage cans found at the respective sampling site. Any other metal can (e.g. tinned food, spray paint or spray hygiene products) was not included in this category.
MetalBottleCaps	The number of beverage metallic bottle caps found at the respective sampling site. Any other metallic non-beverage bottle caps were not included in this category.
MetalAluminumFoil	The number of aluminum foil fragments found at the respective sampling site.
MetalOther	The number of any other item consisting of metal found at the respective sampling site.
GlassBottles	The number of intact glass beverage bottles found at the respective sampling site. Any non-beverage glass bottles were not included in this category.
GlassShards	The number of glass shards (including beverage and non-beverage glass items) found at the respective sampling site. Any other shards (e.g. made from clay) were not included in this category.
GlassOther	The number of any other item consisting of glass found at the respective sampling site.
Paper	The number of any item consisting of paper found at the respective sampling site.
Textiles	The number of any textile (e.g. clothes and fabrics and fragments thereof) found at the respective sampling site.
Rubber	The number of rubber items found at the respective sampling site.
Balloons	The number of balloons found at the respective sampling site.
Other	The number of any other litter item fitting in none of the other categories found at the respective sampling site.
TotalSUP	The total number of single-use plastic items found at the respective sampling site, i.e. the sum of the nine respective categories above.
GrandTotal	The grand total number of litter items found at the respective sampling site, including single-use plastic items.
PercentageSUP	The percentage of single-use plastic items found at the respective sampling site.
WeightPlastic	The weight (in kg) of litter items consisting of plastic (including single-use plastic items) found at the respective sampling site. na = not weighted separately.
WeightTotal_inclPlastic	The total weight of all litter items found at the respective sampling site, including plastic items.
Notes	Any note of interest regarding the respective sampling site.

**Table 6:** Description of data fields of datasheet *5_LitterSources*. The file is organized in rows, each rows represents the data for each sampling site visited by an organization. See below for the description of the evaluation of litter sources.

Data field	Description
ID	A unique identifier for each sampling event.
ID_LitterSources	A unique identifier for this evaluation of litter sources
Residents	Evaluation whether residents contributed to local litter pollution (likely, possibly or unlikely).
Residents_Evidence	Annotated evidence that hinted at the respective source. na = source considered to unlikely contribute to local litter pollution.
Visitors	Evaluation whether recreational visitors contributed to local litter pollution (likely, possibly or unlikely).
Visitors_Evidence	Annotated evidence that hinted at the respective source. na = source considered to unlikely contribute to local litter pollution.
FlyTippers	Evaluation whether fly tippers, i.e. people who dump litter illegally, contributed to local litter pollution (likely, possibly or unlikely).
FlyTippersEvidence	Annotated evidence that hinted at the respective source. na = source considered to unlikely contribute to local litter pollution.
Industry	Evaluation whether industry, e.g. construction industry or a specific company contributed to local litter pollution (likely, possibly or unlikely).
IndustryEvidence	Annotated evidence that hinted at the respective source. na = source considered to unlikely contribute to local litter pollution.
Agriculture	Evaluation whether agricultural activity contributed to local litter pollution (likely, possibly or unlikely).
AgricultureEvidence	Annotated evidence that hinted at the respective source. na = source considered to unlikely contribute to local litter pollution.
Shipping	Evaluation whether shipping, e.g. commercial and recreational shipping activities, contributed to local litter pollution (likely, possibly or unlikely).
ShippingEvidence	Annotated evidence that hinted at the respective source. na = source considered to unlikely contribute to local litter pollution.
Fishing	Evaluation whether fishing contributed to local litter pollution (likely, possibly or unlikely).
Fishing Evidence	Annotated evidence that hinted at the respective source. na = source considered to unlikely contribute to local litter pollution.
Notes	Any note of interest regarding the respective sampling site.
• A report aimed at informing the participating citizen scientists about project outcomes (“Kiessling, Goldchtaub, Walker 2025_Plastic Pirates Nova Scotia Fall 2024 report.pdf”),
• A text file containing the data cards used by the citizen scientists to annotate research data during the sampling activities in the field (“Plastic Pirates Nova Scotia 2024_data cards.docx”).

All personal data has been removed from the dataset for data privacy reasons.

## 4 Experimental Design, Materials and Methods

### 4.1 Background information: The Plastic Pirates citizen science program

The Plastic Pirates program (https://www.plastic-pirates.eu/) is a citizen science initiative involving schoolchildren and their teachers in anthropogenic litter pollution research, with a focus on plastic pollution. Besides the collection of research data, furthering the environmental education and scientific literacy of the schoolchildren are also priorities. The initiative was founded in Germany in 2016 and has since involved >24,000 schoolchildren, who collectively submitted >1300 datasets [10]. The program has been expanded to other European countries. The research methods of the Plastic Pirates are based on the Científicos de la Basura citizen science program, which is active in Latin America [11], and has been adapted to the German and European context. In the fall of 2024, the Plastic Pirates program was implemented in Nova Scotia, Canada.

### 4.2 Participating organizations, citizen science approach and data quality

A total of nine organizations participated in the Plastic Pirates sampling campaign in Nova Scotia between the 10th of October and the 16th of November 2024, among them seven schools, one university engagement program and one non-governmental organization. The collected data therefore represent an evaluation of anthropogenic litter pollution during the fall of 2024 (a repetition of the citizen science initiative to investigate seasonal differences was not possible due to limited finances). A total of 275 people were involved in the project, among them 221 schoolchildren attending the 4th, 5th or 6th grade of school. Collectively, all people involved contributed 635 h of research effort with the purpose to investigate litter density, litter types (Group A) and the composition of individual litter items (Group B; see description of protocols and methods below). Data quality is a frequently discussed aspect in studies involving citizen scientists and especially schoolchildren [12], therefore the Plastic Pirates employ various measures to assess and assure the quality of research data generated within the program [13]. In the case of the implementation of the program in Nova Scotia, established citizen science protocols were used, and a coordinator (TK) attended all samplings in-person and explained the sampling activity to the schoolchildren. This included familiarizing the schoolchildren with the topic of anthropogenic litter pollution, illustrating the research question that each group of schoolchildren addressed, sharing the results of other participating organizations, and also emphasizing that the metric system would be used for measurements. This preparation took approximately 20 min. Subsequently, the coordinator was present throughout the sampling and explained the research protocols (see below). In addition, between one and nine other adults were present during samplings involving schoolchildren, supervising their work after a briefing by the coordinator. After each sampling concluded, the coordinator made sure all datasheets were legible, clarifying ambiguous datasheets with the schoolchildren and teachers. A sampling event concluded with schoolchildren briefly presenting the outcome of their investigation and sharing their impression of litter pollution of the sampling site, also discussing wider implications (e.g. for their daily life or regarding the responsibility for pollution).

### 4.3 Location and characteristics of sampling sites in Nova Scotia

**Fig. 1:**
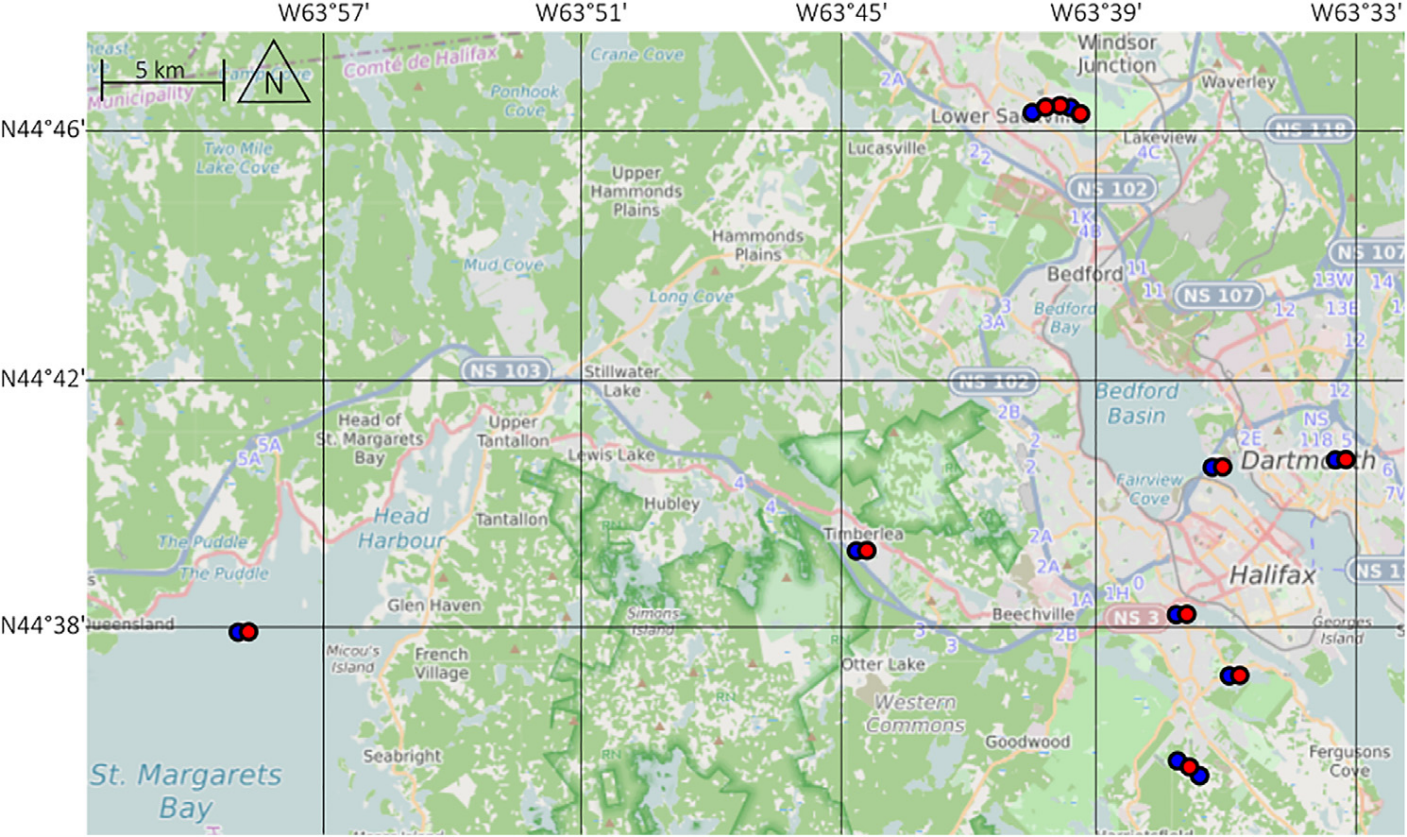
Sampling sites investigated by the Plastic Pirates in Nova Scotia in 2024 with two different protocols (blue = litter density and litter type, Group A; red = litter item composition, Group B) https://umap.openstreetmap.fr/en/map/plastic-pirates-nova-scotia-2024-sampling-sites_1275975 (see the datasheets *1_Metadata* and *2_GroupA_Data* for coordinates).

In total, 20 sampling activities were conducted by the citizen scientists at six different lakes and two ocean sites (Fig. 1). The coordinates for the investigation of litter density and type (Group A) are precise and always reflect the location of the first transect that was established. No precise coordinates for the investigation of litter item composition (Group B) were recorded as the schoolchildren collected litter within a non-confined area. The approximate area of data collection reflected the gathering point of schoolchildren (see datasheet *1_Metadata* and Table 2). Metadata about the characteristics of sampling sites (see Table 2) was assessed by TK, by observing site characteristics in field and using mapping and satellite imagery software such as OpenStreetMap [9] and Google Earth.

### 4.4 Group A: Litter density and litter types

**Fig. 2:**
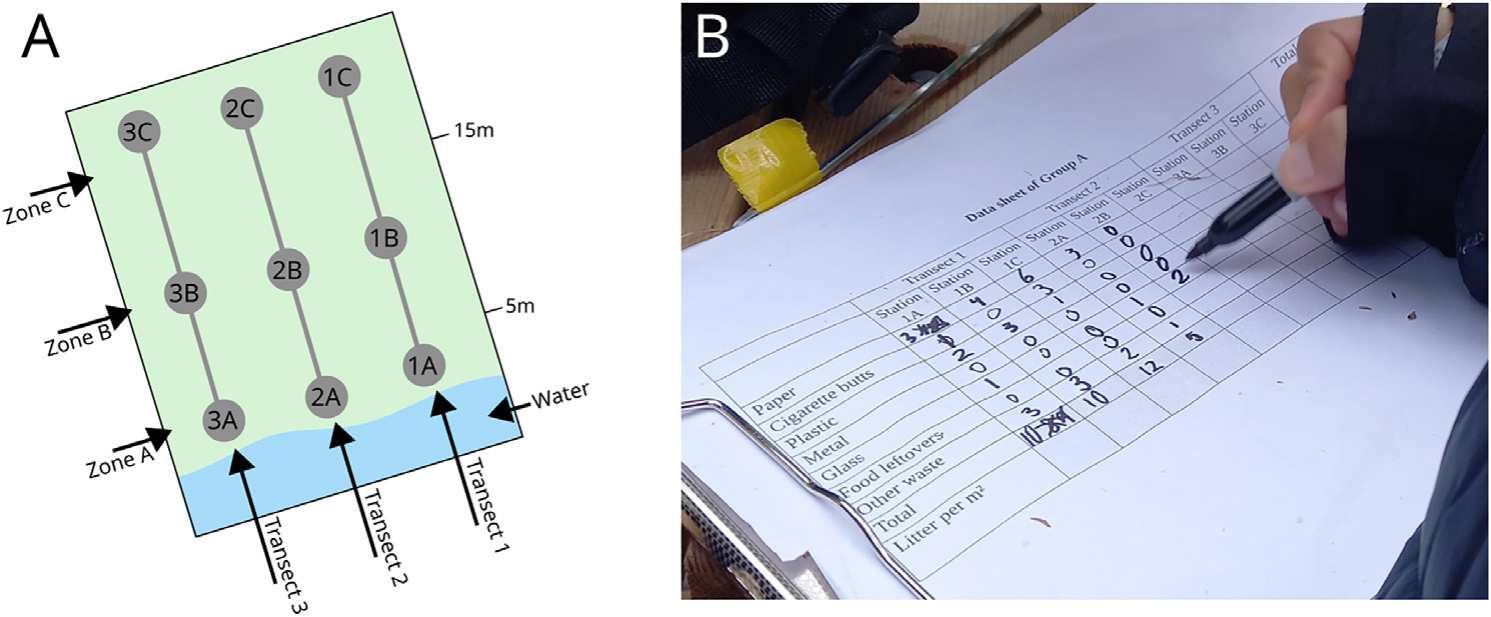
(A) Method to establish transects and sampling stations to investigate litter density and litter types (adapted from Kiessling et al [16]). (B) A schoolchild from Crichton Park School annotated data to investigate litter density and litter types at Lake Banook. Photo by Tim Kiessling, Creative Commons license CC BY 4.0.

To investigate litter density and material composition of litter, the method developed by the Científicos de la Basura program was followed [14], which was adopted to investigate beaches in Europe in the Plastic Pirates citizen science program [15]: Transects were established perpendicular to the shore of the lake or coastline using a tape measure, extending it from the water to the upper part of the shore (Fig. 2A). After, rectangular sampling stations of 3 m x 3 m (9 m²) were established in different zones on the transect. The first station was established within 5 m of the water. The second station was established in between 5 m and 15 m from the water. The third sampling station was established at least 15 m away from the water. Occasionally a station could not be established, for example because of vegetation cover. These stations are annotated in the datasheet and litter quantities are marked with “na” for the respective station. A tape measure, tent pegs and string were used to establish each station, to clearly distinguish between the inside and outside of the sampling area. Subsequently, each sampling station was searched by at least two schoolchildren or volunteers, and all visually identifiable litter items were collected in a bucket. Organic material such as leaves, twigs or algae were overturned to search for litter, but no digging took place to find buried items. Litter items were subsequently sorted into seven different categories: paper, cigarette butts, plastic, metal, glass, food leftover, other waste, in accordance with the Plastic Pirates protocol [15,16]. The data were annotated in a data card (Fig. 2B; and see the file “Plastic Pirates Nova Scotia 2024_data cards.docx”). The total number of established transects and sampling stations, i.e. the investigated surface area of the coast at a sampling site, varied across groups and was dependent on the number of participants, their available time as well as encountered litter quantities. The minimum surface area investigated was 27 m², the maximum 126 m², and the total area at the ten sampling sites amounted to 675 m² investigated with this method (see datasheet *4_GroupB_Data*). Sampling stations on established transects, which could not be investigated were annotated and marked with “na” in the dataset.

### 4.5 Group B: Litter item composition

To investigate litter item composition, the method described by Kiessling et al [17] was followed: Schoolchildren and volunteers collected as many litter items as they could find along the shore within a given time frame (no litter was collected in the sampling area of Group A, investigating litter density). The time frame was dependent on the number of schoolchildren and volunteers and their available time and was approximately 20 to 30 min for most sampling sites. The approximate area investigated was assessed based on inquiring the schoolchildren where they have gone to collect items and subsequently measuring on OpenStreetMap. The collected items were deposited next to a “sorting station”, consisting of a tarp with labels and buckets for the different categories of litter (a total of 24 categories was differentiated, among them nine categories of single-use plastics; Table 5). Subsequently, each item was sorted into the corresponding category. Any item consisting of more than one material was categorized according to the largest fraction of material it contained (for example a glass beverage bottle with metal lid was placed in the “glass beverage bottle” category). Any unknown item or item without a category was either placed in the “other plastic items”, “other metal items”, or “other glass items” categories if the material could be identified or in “other items not fitting into the other categories”. Afterwards, the schoolchildren and volunteers counted the litter items in each category and communicated their result to the person annotating the data (most often a team of two schoolchildren). After the counting, the plastic litter and the total litter was weighted separately and the values annotated in the data card (“Plastic Pirates Nova Scotia 2024_data cards.docx”). At three sampling spots only the total weight of items was registered.

### 4.6 Evaluation of litter sources

To evaluate the sources of litter, the coordinator of the project (TK) followed the method by Kiessling et al [16]: Indicators such as the composition of litter found and nearby sources of litter (such as overflowing waste bins) were used to assess whether the following sources were likely contributing, possibly contributing, or unlikely contributing to local litter pollution of the sampling site: Residents, recreational visitors, fly tippers, industry, agriculture, shipping, or fishing. If a source was evaluated to likely or possibly contribute to local litter pollution, the reasoning for this decision was annotated as well (e.g. which observed litter items indicated the respective source; Table 6).

## Limitations

Overall, 20 sampling activities have been conducted, using two protocols, and seven sites at lakes and two ocean sites have been investigated. A total of 495 litter items were collected and analyzed to assess litter density and type. A total of 3032 items were collected and analyzed to assess litter item composition. This is a comparatively small dataset compared to other citizen science studies investigating anthropogenic litter with the same method, for example the Cíentificos de la Basura in Latin America [7], and the Plastic Pirates in Europe [16,17]. Further, sampling sites were investigated that were mostly well accessible and within the vicinity of participating schools and organizations. Therefore, items typically littered by recreational visitors are likely over-represented in the dataset, whereas pollution by other sources (e.g. illegal dumping, industry, fisheries) was likely underestimated.

## Ethics Statement

We, the authors, confirm that we have read and follow the ethical requirements for publication in Data in Brief and confirm that the current work does not involve human subjects, animal experiments, or any data collected from social media platforms.

## CRediT authorship contribution statement

**Tim Kiessling:** Conceptualization, Methodology, Data curation, Investigation, Writing – original draft, Writing – review & editing, Project administration, Funding acquisition. **Maya Goldchtaub:** Conceptualization, Methodology, Writing – review & editing, Project administration, Funding acquisition. **Plastic Pirates of Nova Scotia:** Investigation, Data curation. **Sinja Dittmann:** Methodology, Writing – review & editing. **Janto Schönberg:** Methodology, Writing – review & editing. **Tony R. Walker:** Writing – review & editing, Supervision, Funding acquisition.

## Data Availability

ZenodoPlastic Pirates Nova Scotia 2024 dataset (Original data).

## References

[1] Kiessling T., Goldchtaub M., Scotia Plastic Pirates of Nova, Dittmann S., Schönberg J., Walker T.R. (Sept. 2025). Plastic Pirates Nova Scotia 2024 dataset. Zenodo.

[2] Beaumont N.J. (May 2019). Global ecological, social and economic impacts of marine plastic. Mar. Pollut. Bull..

[3] Kühn S., Van Franeker J.A. (Feb. 2020). Quantitative overview of marine debris ingested by marine megafauna. Mar. Pollut. Bull..

[4] Landrigan P.J. (Aug. 2025). The Lancet Countdown on health and plastics. Lancet.

[5] Kawabe L.A., Ghilardi-Lopes N.P., Turra A., Wyles K.J. (Sept. 2022). Citizen science in marine litter research: a review. Mar. Pollut. Bull..

[6] Nelms S.E. (Aug. 2020). Investigating the distribution and regional occurrence of anthropogenic litter in English marine protected areas using 25 years of citizen-science beach clean data. Environ. Pollut..

[7] De Veer D. (Nov. 2023). Citizen scientists study beach litter along 12,000 km of the East Pacific coast: a baseline for the International Plastic Treaty. Mar. Pollut. Bull..

[8] Roche J. (Dec. 2020). Citizen Science, education, and learning: challenges and opportunities. Front. Sociol..

[9] OpenStreetMap contributors (2024). OpenStreetMap. https://www.openstreetmap.org/.

[10] Dittmann S. (Aug. 2023). Sharing communication insights of the citizen science program plastic pirates—best practices from 7 years of engaging schoolchildren and teachers in plastic pollution research. Front. Environ. Sci..

[11] Rech S., Macaya-Caquilpán V., Pantoja J.F., Rivadeneira M.M., Campodónico C.K., Thiel M. (June 2015). Sampling of riverine litter with citizen scientists — Findings and recommendations. Environ. Monit. Assess..

[12] Pizzolato L.A.V., Tsuji L.J.S. (Apr. 2022). Citizen science in K–12 school-based learning settings. Sch. Sci. Math..

[13] Dittmann S. (Dec. 2022). Proceedings of Engaging Citizen Science Conference 2022 — PoS(CitSci2022).

[14] Bravo M., De Los Ángeles Gallardo M., Luna-Jorquera G., Núñez P., Vásquez N., Thiel M. (Nov. 2009). Anthropogenic debris on beaches in the SE Pacific (Chile): results from a national survey supported by volunteers. Mar. Pollut. Bull..

[15] J. Schönberg et al., “Plastic Pirates - go Europe! project booklet coasts and beaches.” 2024 [Online]. Available: 10.5281/zenodo.15632712.

[16] Kiessling T., Knickmeier K., Kruse K., Brennecke D., Nauendorf A., Thiel M. (Feb. 2019). Plastic pirates sample litter at rivers in Germany – Riverside litter and litter sources estimated by schoolchildren. Environ. Pollut..

[17] Kiessling T. (June 2023). What potential does the EU single-use plastics directive have for reducing plastic pollution at coastlines and riversides?. Waste Manag..

